# Effect of big eye tuna (*Thunnus* *obesus*) head soup with different colloidal particle size on TG and TC deposition in FFA‐exposed HepG2 cells

**DOI:** 10.1002/fsn3.2092

**Published:** 2020-12-31

**Authors:** Jing Zhang, Liu Lin, Ningping Tao, Zheqing Zhu, Xichang Wang, Mingfu Wang

**Affiliations:** ^1^ College of Food Science and Technology Shanghai Ocean University Shanghai P. R. China; ^2^ School of Perfume and Aroma Technology Shanghai Institute of Technology Shanghai P. R. China; ^3^ Shanghai Engineering Research Center of Aquatic‐Product Processing & Preservation Shanghai China; ^4^ Food and Nutritional Science Program School of Biological Sciences The University of Hong Kong Hong Kong P. R. China

**Keywords:** EPA and DHA, FFA‐exposed HepG2 cells, self‐assembled micro/nanocolloidal, TG and TC accumulation

## Abstract

Micro/nanocolloidal is confirmed as a self‐assembly structure in big eye tuna (*Thunnus obesus*) head soup, and lipids enriched with docosahexaenoic acid (DHA) and eicosapentaenoic acid (EPA) are the major component. In this study, the effect of big eye tuna head soup (BETHS) with different particle size micro/nanocolloidal on lipid accumulation was initially evaluated. The original soup and microfiltration soup (with or without ginger; OGS/OGSG and MFS/MFSG) were prepared firstly. A free fatty acid‐exposed (FFA‐exposed) HepG2 cell model was built using sodium oleic acid (OA) and sodium palmitic acid (PA) (2:1). The triglyceride (TG) and total cholesterol (TC) in the FFA‐exposed HepG2 cells were 8.6 ng/10^4^ cells and 0.6 nM/10^4^ cells, respectively, which were significantly different with control (*p* < .05). Both OGS and OGSG could significantly decline the TG deposition of FFA‐exposed HepG2 cells with 31% and 40% (*p* < .05), and in MFS and MFSG were 23% and 26% (*p* ≥ .05). Meanwhile, OGS inhibited the deposition of TG mainly in 18–24 hr, and OGSG mainly in 12–18 hr. All the BETHS samples showed no inhibition effect on TC deposition (*p* ≥ .05). This research might help to understand the improving activity of natural or traditional food products on metabolic syndrome.

## Graphical abstract

Big eye tuna head soup (BETHS) with 30% ginger had cytotoxicity on HepG2 cells. BETHS with larger micro/nano particles (MNPs) improved TG deposition (*p* < .05). Adding ginger promoted the TG deposition improvement of origin BETHS.

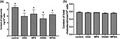

## 1 INTRODUCTION

Big eye tuna (*Thunnus obesus*) is the main target species of Indian and Pacific Ocean (Erdaide et al., 2016). As a kind of major marine fish, tuna can be produced into popular food products including raw sashimi, steak, and canned products. It is reported that the capture of big eye tuna was 472,934 tons in 2017 (Zhang, Tao, et al., 2019). However, there is a large number of tuna by‐products generated during processing, such as head, bone, and skin, which can be 50%–65% of original raw material (Kim et al., 2019; Lu, 2013).

Eicosapentaenoic acid and DHA are two main omega‐3 PUFAs in tuna, and it was reported that tuna roe contains 79.41% DHA + EPA in its PUFA (Restuccia et al., 2015). Our previous studies found that big eye tuna head contains abundant EPA and DHA compared with Atlantic salmon (*Salmo salar*), which is another representative marine fish species (Zhang, Tao, et al., 2019; Zhang, Xu, et al., 2019). EPA and DHA were found had outstanding beneficial effects on cancer, cardiovascular disease, metabolic syndrome, inflammatory disease, neurodegenerative diseases, and liver disease (de Oliveira et al., 2017). Meanwhile, EPA‐ and DHA‐enriched lipids were confirmed that had critical improvement on lipid metabolism (Ding et al., 2016; Gotoh et al., 2009; Mori et al., 2000; Om et al., 2003; Zhang, Tao, et al., 2020; Zhang, Yi, et al., 2020), and majority of them used purified or synthetic lipids.

Soup is considered flavorful, convenient, and easy to absorption. Most of soups are focused on the presence of nutrients substance and molecules responsible for sensorial properties (Sun et al., 2019). However, there are no reports exploring the improvement of lipid accumulation of fish soup ever before. Ginger (*Zingiber officinale Roscoe*) is a kind of common condiment for foods and beverages that is extremely popular in many countries (Makanjuola, 2017). In China, India, Roman, and some other countries, ginger is also used as a medicinal herb for colds, headaches, and other human ailments (Cheng et al., 2011). It is reported that ginger contains abundant polyphenol and flavonoid substances (Ghasemzadeh et al., 2010; Makanjuola & Enujiugha, 2018; Shirin & Prakash, 2010). Ginger could be used to cover the unpleasant smell of meat or fish. In the past, there is no study reported if adding ginger will affect the lipid metabolism improvement activity of fish soup.

Micro/nanocolloidal is a kind of main nutrients organization of natural food (Cheema et al., 2018; Ren et al., 2019). In soups, the nutritional components are gradually migrated into soup from food materials, and those migrated components will interact with each other through the secondary bonds forming a new micro/nanometer scale supramolecular structure (Ke et al., 2017; Qian et al., 2019). Such micro/nanocolloidal is found with a large potential in biological and bioactive application since its outstanding delivery and protection properties (Bollhorst et al., 2017). Meanwhile, we found that those nutrients substances are self‐assembled into micro/nanocolloidal during the boiling process of big eye tuna head soup (Fan et al., 2019; Qian et al., 2019).

In this study, a free fatty acid (FFA)‐exposed HepG2 cells were built using sodium oleic acid (OA) and sodium palmitic acid (PA), and then, the cytotoxicity of big eye tuna with different particle size micro/nanocolloidal was determined. Furthermore, the improvement of lipid accumulation of big eye tuna with different particle size micro/nanocolloidal was investigated and compared with each other. This work will help to better understand the effect of big eye tuna head soup on lipid deposition, and provide a theoretical basis for the utilization of fish by‐products and development of soup.

## 2 MATERIAL AND METHODS

### 2.1 Materials

Half of big eye tuna head (number: 30; length: 28.0 ± 3.0 cm; weight: 1.55 ± 0.23 kg) was purchased from Xiang Xiang Food Co., Ltd. Originally, the big eye tuna was captured from Pacific and Indian Ocean. Ginger (*Rhizoma Zingiberis Recens*) was purchased from Nonggongshang supermarket at Guzong Road, Pudong New Area, Shanghai City, and produced from Laiwu City, Shandong Province. Organic solvents were purchased from Shanghai ANPEL Scientific Instrument CO., Ltd. Oil red O was purchased from MACKLIN. Cell counting kit‐8 (CCK‐8) was purchased from Dojindo Molecular Technologies, Ins. Hematoxylin and formalin were purchased from Solarbio. Minimum Eagle's medium (MEM) and fetal calf serum (FBS) were purchased from Thermo Fisher Scientific. Penicillin‐streptomycin was purchased from Hyclone. OA, PA, and low fatty acid bovine serum albumin (BSA) were purchased from Sigma‐Aldrich.

Pure CO_2_ (99.99%) was used from Shanghai Li Dan Industrial Gases Ltd.

### 2.2 Preparation of big eye tuna head soup

The deep‐frozen big eye tuna heads were defrosted using running‐water thawing method. Then, the heads were cut into small pieces with the size of 5 × 3 × 2 cm^3^ and washed with drinking water. The pieces were drained before weighing. After that, 400 ± 2 g pieces was fried with 40 ± 1 g soybean oil (first grade) for 40 s at 120°C in a pan and then boiled stew with medium heat. Briefly, fried big eye tuna head pieces and 3,200 ml drinking water were put into a stainless steel pot and stewed in a soup model with the multi‐function introduction cooker (C21‐WT2112T). The cooking process was as follows: boil at 97 ± 2°C for 30 min and keep the temperature of 90 ± 2°C for 150 min. Meanwhile, the big eye tuna head soup with ginger was prepared as follows: The ginger was cut into small pieces with the size of 2 × 1 × 0.5 cm^3^ and cooking with fried big eye tuna head pieces, and the cooking process was the same as the big eye tuna head soup without ginger.

Original soup (with or without ginger; OGS/OGSG) and microfiltration soup (with or without ginger; MFS/MFSG) were prepared furtherly. OGS and OGSG were obtained using gauze to filter the cooked soup. MFS and MFSG were prepared as follows: The cooked soup was centrifuged at 5,000 rpm for 10 min, and the supernatant was filtered using 0.45‐μm membrane. The filtrates above were collected and stored at −30°C.

### 2.3 Cell culture and treatment

HepG2 cells were cultured in MEM supplemented with 5% FBS and penicillin‐streptomycin at 37°C in an atmosphere containing 5% CO_2_. Then, 70%–80% influent cells were seeded into 96‐well plates and 6‐well plates.

Three different groups were settled: (a) control group: cultured in the stated medium; (b) FFA‐exposed HepG2 cells group: pretreated by OA/PA (2:1) with different concentration for 24 hr and then cultured in stated medium; (c) pretreated by OA/PA (2:1) and then incubated with big eye tuna head soup samples for 24 hr.

### 2.4 Cell viability assay

The effects of OA/PA (2:1) on HepG2 cells’ viability and big eye tuna head soup on FFAH cells’ viability were analyzed by Cell counting kit‐8 (Dojindo Molecular Technologies) according to the manufacturer's recommendation.

### 2.5 Staining using Oil red O

HepG2 cells were washed with PBS and then fixed with formalin for 10 min at room temperature and then washed with PBS and 60% isopropanol separately. Then, the cells were stained by Oil red O solution for 15 min at room temperature and then washed with 60% isopropanol for 10 s. Next, the cells were secondly stained with hematoxylin for 1 min and then washed with distilled water. Then, the stained cells were observed using an EVOS XL Core Imaging system (Thermo Fisher Scientific).

### 2.6 Measurement of the intracellular triglyceride and total cholesterol

In this study, the intracellular TG and TC of FFA‐exposed HepG2 cells were evaluated to determine the inducing concentration of OA/PA (2:1), and to compare the effects of improve lipid deposition of big eye tuna head soup samples. The intracellular triglyceride (TG) and total cholesterol (TC) were evaluated using two assay kits (Solarbio).

### 2.7 Statistical analysis

All scientific experiments were repeated at least three times. Results were presented as mean ± *SD*. All statistical analyses were done using SPSS version 10.0 software (SPSS Institute). *p* < .05 and .01 was considered statistically significant and extremely significant, respectively.

## 3 RESULTS AND DISCUSSION

### 3.1 Induction of HepG2 using free fatty acids

The OA/PA (2:1) was used as inducer to build HepG2 model cells in this study. The most proper inducing condition was determined according to the effect of different concentration inducer on HepG2 cells’ viability, Oil red O staining results, and the intracellular TG and TC content.

**FIGURE 1:**
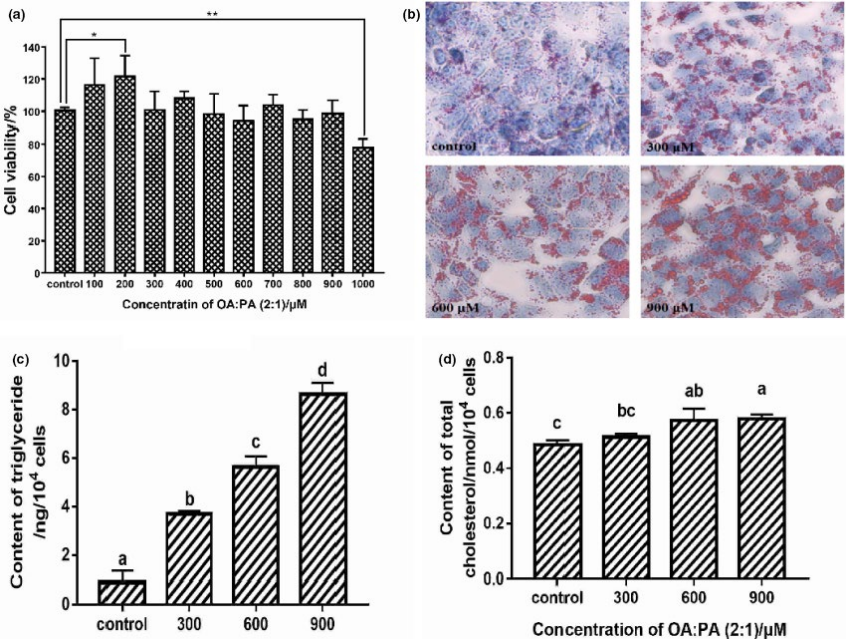
(a) Effect of different concentration OA/PA (2:1) on the viability of HepG2. (b) The dying results of HepG2 using Oil red O. (c) The TG accumulation in HepG2 induced by different concentration of OA/PA (2:1). (d) The TC accumulation in HepG2 induced by different concentration of OA/PA (2:1). *, ** and ^a–c^ mean significant difference between groups (*p* < .05 or .01), the same below

It was showed that the OA/PA (2:1) in 100–900 μM did not decline the cell viability, and all the cell viability was higher than 90% compared with control group (Figure 1a). The relative cell viability of 200 μM OA/PA (2:1) group was significantly higher than control group (*p* < .05). In this study, the low fatty acid (*p* ≤ .02) BSA was used as the carrier of OA/PA (2:1) to induce FFA‐exposed HepG2 cells model. As we know, there are 583 amino acid contained in BSA, and it is speculated that BSA might improve the HepG2 cells growing. Luo and others found that BSA could enhance the relative cell viability of HUVEC cells compared with control group, and the combination with BSA will decrease the cytotoxicity of heme (Luo et al., 2020). However, the cell viability was only 78% (*p* < .01) when the concentration of OA/PA (2:1) was 1,000 μM. OA and PA are two kinds of fatty acid sodium, and it was found that sodium palmitate was able to induce lipoapoptosis in L02 and HepG2 cells to reduce their relative cell viability (Cao et al., 2014). With the increase in concentration of PA, the lactate dehydrogenase (LDH) release and caspase activity in HepG2 cells were markedly enhanced (Luo et al., 2012), and it is speculated that high concentration OA/PA (2:1) might induce the apoptosis of HepG2 cells. Therefore, the OA/PA (2:1) with 100–900 μM concentration can be used to build FFA‐exposed HepG2 cells model.

Intracellular lipid accumulation was visually observed by Oil red O staining, and the 300, 600, 900 μM OA/PA (2:1) were selected to treat HepG2 cells to observe the Oil red O staining results. As shown in Figure 1b, obvious red lipid droplets existed in the three FFA‐exposed HepG2 cells groups compared with control group. Meanwhile, with the increase in OA/PA (2:1) concentration, it seems that the red lipid droplets also increased. Hence, the Oil red O staining results indicated that OA/PA (2:1) could effectively induce the accumulation of lipid in HepG2 cells.

The contents of TG and TC of FFA‐exposed HepG2 cells were determined using assay kits. As shown in Figure 1c, the TG of 300 μM group was significantly increased (*p* < .05) compared with control group, which was enhanced to 3.7 from 0.9 ng/10^4^ cells. The 600 and 900 μM groups were also significantly higher than 300 μM group (*p* < .05), and there was no significant difference between 600 and 900 μM (*p* ≥ .05), which were 5.6 and 8.6 ng/10^4^ cells, respectively. For the determination of TC accumulation, the 600 and 900 μM groups were significantly higher than the control group (*p* < .05), and the TC of 600 and 900 μM groups was about 0.6 nM/10^4^ cells (Figure 1d). Considering the cytotoxicity, Oil red O staining results, and accumulation of TG and TC, the 900 μM OA/PA (2:1) was used to build the FFA‐exposed HepG2 cells in the following study.

### 3.2 Effect of OGS/OGSG and MFS/MFSG on cell viability of FFA‐exposed HepG2 cells

**FIGURE 2:**
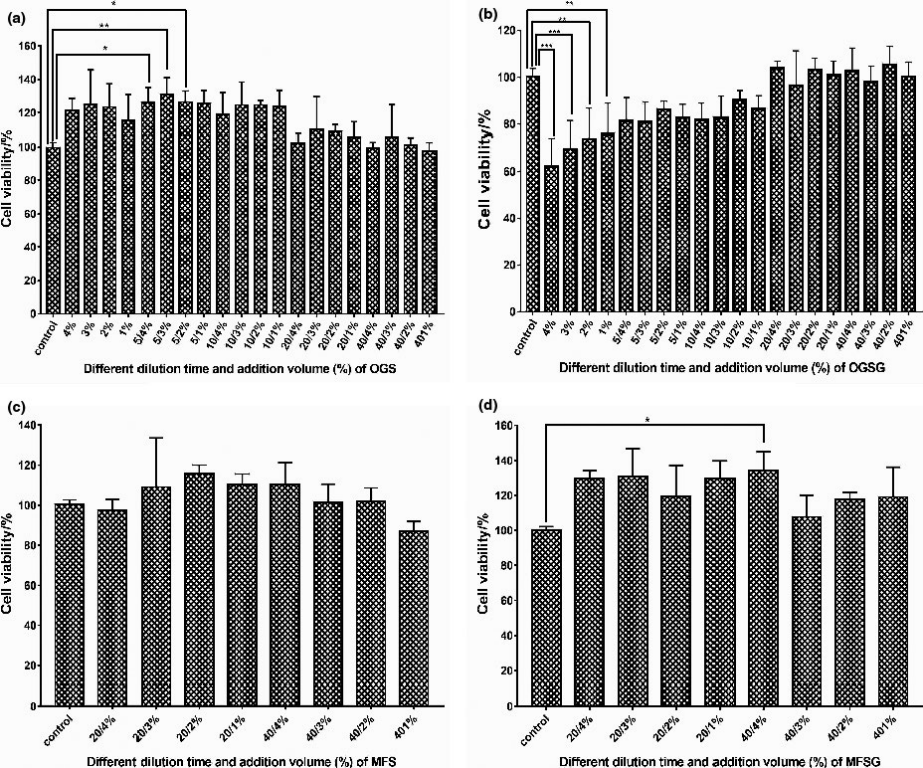
Effect of different dilution time and addition volume of (a) OGS, (b) OGSG, (c) MFS and (d) MFSG on FFA‐exposed HepG2 cells

In order to select the proper addition volume of big eye tuna head soup, the cytotoxicity of OGS/OGSG and MFS/MFSG in FFA‐exposed HepG2 cells was evaluated. Various dilution time (1, 5, 10, 20) and addition volume (1%, 2%, 3%, 4%) were settled in this study. The cell viability is shown in Figure 2a; all the OGS groups were higher than 90%, which means that OGS has no cytotoxicity on FFA‐exposed HepG2 cells. When the dilution time was 1, 5, and 10, their cell viabilities were higher than control, and three groups (5 dilution time/4% addition volume, 5/3%, 5/2%) were significantly (*p* < .01 or .05) higher than control. Meanwhile, the cell viability of 20 and 40 dilution time groups was gradually declined to 100%, and they had no significantly difference with control (*p* ≥ .05). It was reported that the big eye tuna head soup is rich in nutrients including lipid, protein, and sugar (Qian et al., 2019), and these substance might improve the growth of FFA‐exposed HepG2 cells. With the increase in dilution time, the content of OGS in medium was decreased, and the improvement was gradually declined to 100%.

The effect of OGSG on cell viability of FFA‐exposed HepG2 cells was opposite to OGS. The cell viability presented a gradually rising trend with the increase in dilution time and decrease in addition volume of OGSG, and the 1, 5, and 10 dilution time groups were lower than 90% (Figure 2b). When the high dilution time (20 and 40) OGSG was added in the medium, the cell viability was closed to or higher than 90%, and they have no significant difference with control (*p* ≥ .05). The difference between OGS and OGSG might be caused by the addition of ginger. In the boiling process, the polysaccharide, polyphenolic, and curcumin might gradually migrate into the big eye tuna head soup. However, it was reported that the ethanol extract of ginger could inhibit the growth of HepG2 cells by stimulating the mechanism of apoptosis (Mahomoodally et al., 2019; Wang et al., 2019). In this study, the content of ginger dissolution substance in OGSG with low dilution time (1, 5, and 10) might be relatively high, and that might reduce the cell viability of FFA‐exposed HepG2 cells. With the increase in dilution time and decrease in addition volume, the content of ginger dissolution substance in OGSG was also declined, and its effect on the cell viability of FFA‐exposed HepG2 cells was weaken.

Furtherly, the cytotoxicity of MFS and MFSG in FFA‐exposed HepG2 cells was determined (Figure 2c,d). Based on the results above, two dilution time (20 and 40) and four addition volumes (1%, 2%, 3%, and 4%) were settled in this part. As shown in Figure 2c,d, the cell viability of all MFS and MFSG groups was higher than 90%, which means the MFS and MFSG with high dilution time will not affect the viability of FFA‐exposed HepG2 cells. Except the MFSG with 20 dilution time and 4% addition volume group, all the other groups had no significant difference with control (*p* ≥ .05).

In summary, all the OGS/OGSG and MFS/MFSG will be diluted 20 time, and their addition volume will be 4% in the following studies.

### 3.3 Effect of OGS/OGSG and MFS/MFSG on TG and TC deposition in FFA‐exposed HepG2 cells

Nowadays, chronic disease is a major challenge in Human society, and nonalcoholic fatty liver disease (NAFLD) is an increasingly serious health problem, which means a pathological syndrome that is not caused by excessive alcohol consumption, and characterized by liver cell steatosis and lipid accumulation. The pathogenesis of NAFLD is not clear. At present, it is generally believed that TG accumulation and imbalance of apolipoprotein synthesis are important mechanisms causing nonalcoholic fatty liver disease. However, there are no effective drugs for treating this chronic syndrome as so far. Some lipid‐lowering drugs temporarily transport lipids into the liver, and decrease blood lipids with the increase in liver TG accumulation, which will aggravate the formation of NAFLD (Wang, 2012; Wang et al., 2010). Therefore, from the perspective of diet, the prevention and reduction of liver lipid deposition as much as possible may provide a new solution for improving this chronic syndrome.

**FIGURE 3:**
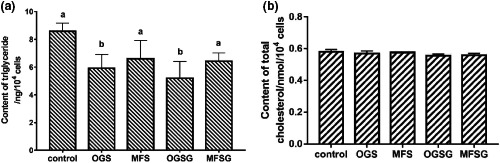
Effects of OGS, OGSG, MFS and MFSG on the accumulation of (a) TG and (b) TC in FFA‐exposed HepG2 cells

As shown in Figure 3a, the content of TG in FFA‐exposed HepG2 cells treated with OGS and OGSG was significantly reduced (*p* < .05), and there was no significant difference between control, MFS, and MFSG groups (*p* ≥ .05). Compared with the control group, the OGS, MFS, OGSG, and MFSG reduced the TG content in FFA‐exposed HepG2 cells by about 31%, 23%, 40%, and 26%, respectively. It is worth noting that the effect of reducing the TG deposition of the original soup is significantly higher than that of the microfiltrate (*p* < .05), and the addition of ginger will strength the effect of improving TG deposition from the declining volume.

Total cholesterol contains the sum of cholesterol in all lipoproteins, including free cholesterol and cholesterol esters. From Figure 3b, compared with the control group, the TC contents of the four experimental groups were 98%, 99%, 96%, and 96% of the control group, respectively. There was no significant difference between the experimental group and the control group (*p* ≥ .05). However, from the decreasing content, the effect of big eye tuna head soup with ginger on intracellular TC is more significant, which has a similar trend with TG.

The improvement of n‐3 PUFA on lipid metabolism has been confirmed. Lee et al. (2015) studied the effect of Antarctic krill oil on cellular TG content by establishing a HepG2 high‐fat cell model, and found that Antarctic krill oil can significantly reduce TG deposition in cells. In addition, the researchers used mice to build high‐fat animal models and fed different sources of EPA/DHA‐rich diets. They found that compared with the control group, the deposition of TG and TC in the serum and liver of the experimental group were significantly improved (Liu, 2014; Wang, 2016). The regulatory mechanism of n‐3 PUFA such as EPA and DHA on lipid metabolism can be summarized as that they can inhibit mRNA expression of genes related to lipid metabolism and synthesis such as SREBP‐1C, ACC‐1, FAS, and SCD‐1, and promote AMPK and PPAR‐ α, CPT‐1, DGAT, and other lipid metabolism degradation‐related gene mRNA expression (Wang, 2016; Zhang, Xu, et al., 2019). At the same time, it was found that different n‐3 PUFAs have different regulatory effects on abnormal lipid metabolism. Both EPA and DHA lipids have a role in improving lipid metabolism, but EPA‐type lipids are more effective in inhibiting body fat accumulation and improving lipid metabolism, and DHA‐type lipids are better in regulating blood pressure (Liu, 2014). Our previous study found that DHA is the most important n‐3 PUFA in big eye tuna head soup, which is several times the content of EPA (Zhang, Tao, et al., 2020; Zhang, Yi, et al., 2020). It is speculated that the lower content of EPA of the soup samples might contribute to the not obvious TC lowering effect.

Meanwhile, the results showed that the lipid deposition‐improving effect of the OGSG was slightly higher than OGS, but there was no significant difference (*p* ≥ .05). Two possible reasons might contribute to that difference. Firstly, our previous study found that adding ginger could significantly improve the lipid oxidation in big eye tuna head soup, and significantly increase the content of n‐3 PUFA. Therefore, the higher content of EPA and DHA in the OGSG and MFSG might more effectively improve the lipid accumulation. In addition, the ginger phenols, flavonoids, and gingerol might dissolve into the soup during the cooking process of the big eye tuna fish head soup, and these substances have been shown to reduce lipid deposition with various extents (Gao, 2016; Hashem et al., 2017; Sung et al., 2019); they may also contribute to the improvement of TG deposition of OGSG and MFSG in this study. However, because the big eye tuna fish head soup is a very complex food system with a variety of nutrients, we are only initially exploring its lipid deposition improving effect, and the specific components and mechanisms need further study.

For the big eye tuna head soup with different sizes colloidal particles, the TG‐reducing activity of the original soup is better than that of the microfiltrate (*p* < .05). In the early stage, the microstructure of micro/nanocolloidal particles in big eye tuna head soup was investigated using laser scanning confocal microscopy and inverted optical microscope. It was found that the center of the colloidal particles is mainly TG, and the outer membrane is mainly phospholipids, and few protein particles are embedded (Fan et al., 2019; Qian et al., 2019). Meanwhile, EPA and DHA of big eye tuna head soup were found mainly existed in triglyceride and phospholipid molecules. It is speculated that during the big eye tuna head soup cooking process, the micro/nanocolloid particles with larger particle size can protect more of the EPA and DHA encapsulated therein, and avoid lipid oxidation and further degradation reactions. Therefore, in addition to retaining more nutrients in the original soup, having a larger particle size of micro/nanocolloid particles may be the main reason for this light difference.

**FIGURE 4:**
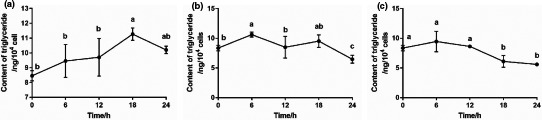
Monitoring of effect of OGS and OGSG on the accumulation of TG in 24 hr. (a) control, (b) OGS and (c) OGSG

By analyzing the effect of big eye tuna head soup on the accumulation of TG and TC in FFA‐exposed HepG2 cells, it was found that OGS and OGSG have significant improvement effect on TG deposition and no significant effect on TC deposition. Therefore, the process of big eye tuna head soup improving the TG accumulation in FFA‐exposed HepG2 cells was monitored. Since the OGS and OGSG declined the TG accumulation more effectively, the OGS, OGSG, and an FFA‐exposed HepG2 cells control group were settled, respectively, and the intracellular TG contents of the three groups were determined every 6 hr for 24 hr. As shown in Figure 4a, through the 24‐hr process monitoring, it can be seen that the TG content of the control group increased slowly within 24 hr and with slight fluctuations during the period. OGS was similar to that of the control group in the first 6 hr and then declined and kept lower than the control group and then significantly declined in 18–24 hr (*p* < .05; Figure 4b). To the TG level of OGSG, it has a relatively obvious increase in the range of 0–6 hr, followed by a significant decrease in the range of 12–18 hr (*p* < .05); the decline within 18–24 hr tended to be flat (Figure 4c).

It is speculated that since the FFA‐exposed HepG2 cells were in the growth phase in the early stage, and the intracellular TG content will also increase, the experimental groups and the control group showed different extents of increase. When the growth of cells decreased until stopped, various nutrients in the cells may be consumed, and the content of nutrients will also stop rising. After that, the control group was only treated with the regular culture medium without soup samples, so it had a small fluctuation in its TG content. In the OGS group, the EPA and DHA of OGSG might begin to regulate various genes related to lipid metabolism in the cells and lead to the reduction of TG deposition. For the OGSG group, the substances possibly migrated from ginger might promote the reduction of TG deposition (Lai et al., 2016; Li et al., 2018; Rahimlou et al., 2016; Wang et al., 2017), which showed a different downward trend with the OGS group.

## 4 CONCLUSION

An FFA‐exposed HepG2 cells model was built by 900 μM OA/PA (2:1) with 24‐hr induction. The Oil‐red O staining result showed obvious difference of the existence of lipid droplets between the FFA‐exposed HepG2 cells and control, and the concentration of TG and TC in FFA‐exposed HepG2 cells was 8.6 ng/10^4^ cells and 0.6 nM/104 cells, which were significantly different with control croup (*p* < .05). OGSG showed more cytotoxicity compared with OGS (*p* < .05), and that might attribute to the substance which migrated from ginger.

Both OGS and OGSG significantly declined the TG deposition of FFA‐exposed HepG2 cells with 31% and 40% (*p* < .05), and the reduced contents of TG in MFS and MFSG were 23% and 26% (*p* ≥ .05). According to the TG content monitoring during 24 hr, OGS inhibited the deposition of TG mainly in 18–24 hr (*p* < .05), and OGSG mainly from 12 to 18 hr (*p* < .05). For TC accumulation, all the big eye tuna head soup samples showed no inhibition effect (*p* ≥ .05). This study provides an initial evaluation of lipid accumulation inhibition of fish soup, and that might help to investigate the improvement activity of natural or traditional food products on lipid metabolism.

## 5 ETHICAL REVIEW

This study does not involve any human or animal testing.

## CONFLICT OF INTEREST

The authors declared that they have no conflicts of interest to this work.

## AUTHOR CONTRIBUTIONS

Jing Zhang conceptualized the data, designed methodology, conducted the experiment, wrote and edited the original manuscript. Liu Lin designed methodology, conducted the experiment, wrote and edited the original manuscript. Ningping Tao conceptualized the data, designed methodology, reviewed and edited the manuscript. Zheqing Zhu conducted the experiment. Xichang Wang supervised the data and administered project. Mingfu Wang supervised the data, reviewed and edited the manuscript.
